# Clinics from the Brain Physiologist's Viewpoint

**Published:** 1930

**Authors:** R. J. A. Berry

**Affiliations:** Director of Medical Services, Stoke Park Colony


### Clinics from the Brain Physiologist's Viewpoint.

By R. J. A. BERRY, M.D., F.R.C.S., F.R.S.E.,
*Director of Medical Services, Stoke Park Colony.*

As one who has had considerable experience of mental work amongst normal and abnormal children, first in the special clinic which I had the privilege of instituting in the Children's Hospital, Melbourne, and more latterly at Stoke Park Colony, Bristol, I am naturally in entire agreement with the opener of this discussion, Dr. Posthuma, in her appeal for the establishment of Child Guidance Clinics in this country. She has given us an admirable account of the American models of such clinics, and her account I can confirm from my own experiences in the United States. Where, possibly, I may differ from Dr. Posthuma, assuming that I have not misunderstood her, is that I do not regard the American model as the best to be followed or established in Great Britain, because I believe that we can do much better than the Americans. Those of you who are at all familiar with the history of medicine in Great Britain and the United States of America will know just as well as I do that, with very few exceptions, medical education in the States was, until quite recently, in a backward condition, consequent on the persistence, into quite recent times, of the American proprietary medical school, the products of which were not infrequently medical cranks, quacks, and ignoramuses, who bought their medical degrees for a few dollars. Notwithstanding that the Americans have swept away most of these places, and erected in their stead splendid medical schools, hospitals and laboratories which command one's whole-hearted admiration, the cloven hoof of the faddist is still everywhere visible in the United States of to-day. There is much to admire in the United States, there is much we might, with advantage to ourselves, copy and emulate—Child Guidance Clinics among them—but there is also much to be avoided. Confronted, as we are, with the distinct possibility of the Americanization of Europe and Empire, it behoves us to walk carefully in the selections we make from the good and the bad in the vast United States of America.

Dr. Posthuma has very correctly made it clear that, in their Child Guidance Clinics, the Americans talk much of psychology, psychiatry, psychoses, infantile emotional strains, and other resonant words and phrases, whilst the final diagnosis is established in a species of Rotary Club round-table conference. The natural and inevitable result is that the main factor is missed or largely overlooked, and that is, that the child is a growing developing organism with an expanding brain, and that developing brain is the key to the whole problem of the child and his reactions. I have never yet met in any American Child Guidance Clinic anyone on the staff who possessed what I, at least, regard as an adequate knowledge of brain physiology. That this should be so in the United States causes no surprise, but that we in Great Britain should look to American psychologists for guidance, rather than to our own splendid galaxy of brain physiologists — Sherrington, Shaw Bolton, Watson, Albert Wilson, Mott, Tredgold, and very many other equally distinguished mental specialists—is surely an unfortunate instance of the prophet being without, if not honour, at least an adequate following in his own country.

In our Child Guidance Clinic work at the Children's Hospital in Melbourne we followed, or attempted to follow, British teaching rather than American, for the very simple reason that we believe British neurology and brain physiology far in advance of anything in the States, and we were entirely satisfied with the results. After a long laboratory, practical, clinical and scientific investigation, we eventually put into operation at the Children's Hospital a special type and mode of examination of every child referred to the clinic by the medical staff. These measurements and observations, in which the Binet tests form only a small part, and are even then employed, not as a measure of intelligence, which they are not, but as an indication of what has gone into the child's brain from the receptor side of its nerve mechanism, whilst the Porteus tests are similarly utilized as a check on the child's effector output, are all devised with the express object of determining, where possible, the relative state of development of the child's brain, because his reactions depend upon and vary with that development. These methods have been so fully described by me elsewhere, and are available to all in book form, that I shall not here describe them. They will, however, be put into active operation at Stoke Park within, I hope, the next three months, and as you will then be able to see them for yourself, we shall be quite content to stand or fall by the results, and by your judgment of those results.

Speaking broadly, every human being depends for his mentality on two great factors—heredity and environment. There is to-day no room for discussion and squabbles as to the relative importance of these two factors, because we now know the truth. Both play their part in developing the brain up to the necessary standard of a normal reaction to the environment. Again, and equally broadly, all human beings may be divided into the unsane, the sane and the insane, or, in more accurate scientific language, the aments, the normals, and the dements. In our physiological development those of us who live to a sufficiently ripe old age pass through all three stages. We commence life as senseless automata, rapidly unfold to Nature's greatest triumph — the normal human brain, and gradually undergo sexual, cerebral and somatic dissolution.

As regards the potent influence of heredity in determining an adequate supply of cells in the child's brain, there is to-day no room for doubt. Every study shows the appalling consequences of a rotten heredity, not only on the brain growth of the child, but in the terrible social, economic and national results which follow such degrees and stages of unchecked amentia. These unfortunate products of heredity — the degenerates with too few brain cells to give those reactions to their human environment described and regarded as normal by normal people—cannot adjust themselves to the social conditions around them. With an ineffective pyramidal-cell layer of control over those animal instincts present in all of us, but which we like to pretend are not, they freely propagate their kind, and have, on an average, twice or three times as many children as do the normal. They therefore continually add to our burdens of pauperism, degeneracy and crime. Surely, then, it is of the first importance to ascertain, if possible, the actual state of development of the brain of the growing child, and which way he is likely to go.

It seems to be forgotten in most of the American Child Guidance Clinics that at birth the child's brain is largely in a neuroblastic condition, and that these neuroblasts have to be converted into fully-matured and medullated neurons before we can attain even a moderate degree of human intelligence. Sherrington's work points to the fact that this conversion of neuroblast into neuron results from incoming nerve impulses from entero-ceptive, proprio-ceptive, and especially extero-ceptive senses. Expressed much more simply, this is where environment enters into the problem. These impulses are perpetually pouring into the child's brain, constantly modifying the neuronic pattern of that brain, and just as constantly altering the mentality and the nature of the reactions to the environment. So long as educationists continue to teach that half truth, or more accurately one-fifth of the truth, that we have only five special senses, instead of about twenty-five, just so long shall we continue to fail to realize the vast importance of environment and education in modifying mentality. And here undoubtedly we have much to learn from the United States, for their social service hospital system commands one's wholehearted admiration. After my return from the States I put it into operation at the Children's Hospital, Melbourne, so far as our very modest financial resources permitted, and the following case from that hospital will serve to illustrate the significance and effects of heredity and environment, and the uses of an intelligent social service :—

A boy of very doubtful mentality was under my observation from about the age of 11 to 14 years. His I.Q. by the Binet and Porteus tests was a little below normal, but he was undersized, microcephalic, and of bad heredity, and I was, therefore, notwithstanding his educational tests, strongly suspicious of high-grade amentia. However, in view of his fair educational record, we gave him the benefit of the doubt, but kept him under observation. In November, 1928, after the onset of puberty, he again appeared at my clinic, with a report from the police of grave sexual misbehaviour of criminal type. Powerful new impulses were now passing into and profoundly altering his brain, so the diagnosis soon became certain. On medical grounds it became equally certain that the neurological facts were of greater import than the educational, for they showed a lack of development of the controlling supra-granular pyramidal-celled layer of the cortex, and were now of much more significance than the more educational Binet tests. A prognosis of potential dangerous criminality, unless an adequate supervision could be exercised, was made, and the Social Service system was set in operation. My assistant was asked to examine and report on the home surroundings and the nature of the environmental influences to which the boy was subjected. These were uniformly bad. Both the parents were mentally deficient, the home environment was deplorable, and it became of the utmost importance—if the boy was to be saved for society—to remove him to a better and different environment. Arrangements were made to have him taken into a home for such boys under the care of one of the Anglican churches, but the mother refused point-blank, not because of any great or undue affection for the boy, but because she considered him to be of a wage-earning age. Within three weeks of this effort the boy murdered his younger brother, and thus established the accuracy of both diagnosis and prognosis, and proved the dual influence of heredity and environment.

It is true that one swallow does not make a summer, but, fortunately or unfortunately, I can give you not one proof but many of the absolute necessity of studying, not so much the emotions and fads of the child, as the Americans would have us believe, but the state of development of the child's brain, in accordance with the teachings of the best and greatest of our English brain physiologists.

All my experience leads me, therefore, strongly to support Dr. Posthuma in her eloquent appeal for Child Guidance Clinics, but that experience also leads me to believe that embryology and physiology should be our guiding stars, rather than the -ologies and -isms of a non-scientific and often non-physiological psychology. The children of to-day are the real wealth of the nation. Is then their future to depend on the unverified opinions of an amateur, superficial, and bungling decade, or is it to be the product of scientific research and knowledge ?

